# High Absorption and Elasticity of a Novel Transgenic Silk with Egg Case Silk Protein from *Nephila clavata*

**DOI:** 10.3390/ijms252312793

**Published:** 2024-11-28

**Authors:** Yichen Wang, Yuhang Lin, Yongkang Luo, Di Zeng, Haibo He, Tianfu Zhao

**Affiliations:** 1 College of Sericulture, Textile and Biomass Science, Southwest University, Chongqing 400715, China; wangyichen1998@126.com (Y.W.); linyuhang1223@163.com (Y.L.); 13304038512@163.com (Y.L.); dehua1105991604@gmail.com (D.Z.); hhb_swu@126.com (H.H.); 2 State Key Laboratory of Resource Insects, Chongqing 400715, China

**Keywords:** spider egg case filament protein of *Nephila clavata*, genetically modified silkworms, *piggy*Bac, cocoon silk performance

## Abstract

Spider silk is part of a special class of natural protein fibers that have high strength and toughness: these materials have excellent comprehensive properties that are not found in other natural fibers (including silk) or most synthetic fibers. Spider egg case filaments have good hardness, can resist water, can protect spider eggs from external threats, have a significantly high initial modulus and high moisture absorption rate, and are expected to be used as a new generation of environmentally friendly natural polymer fibers and biomaterials. However, spiders are predatory and difficult to rear in large numbers, and it is also difficult to obtain spider egg case filaments in large quantities. Silkworms and spiders have a similar spinning system, and the use of transgenic technology in silkworms can obtain stable and high-yield exogenous gene proteins for a long time, representing an ideal bioreactor for the production of spider silk. In this study, the eukaryotic bioreactor and *piggy*Bac transposon system were employed to recombinantly introduce the egg case silk protein of *Nephila clavata* (*Nc*-CYSP1) into the silkworm in the silkworm heavy-chain expression system. The results revealed that the silk glands produced a new type of transgenic silk with a significantly high initial modulus and high moisture absorption. In summary, this study provides an experimental reference for future research on the large-scale production and application of spider egg case filamentous protein, with great application prospects in the development of new environmentally friendly materials.

## 1. Introduction

Spiders constitute a large and diverse group and belong to *Arthropoda*, *Arachnida*, and *Araneae*. The spider studied in this paper was *Nephila clavata*, which belongs to *Tetragnathidae* and *Nephila*. Spider silk, also known as “biosteel” [1], has many excellent characteristics, such as high strength, elasticity, flexibility, extensibility, and fracture resistance. Spider silk is light, resistant to ultraviolet rays, biodegradable, etc., and has garnered extensive attention in chemistry, biology, materials science, and other disciplines as an animal silk fiber [2]. Spiders produce many different filaments throughout their lives, each originating from a distinct filament gland. Orb-weaving spiders, for example, produce seven different specific silk proteins during their lives [3]. Each spider’s silk has unique material properties, including varying degrees of viscosity, strength, hardness, scalability, etc.

Spider egg case filaments, known as tubuliform spidrone silk or cylindrical silk, are produced exclusively by female spiders at the end of their lives. These filaments encapsulate the eggs in a protective shell, shielding them from environmental hazards, bacteria, moisture, etc. [4,5,6]. Spider egg case filaments, mainly composed of CySp1 and CySp2, feature ~24 tandem repeats in the core region of *Nephila clavata*, rich in serine and GVGAGASA motifs, distinct from other silk types such as GPGGX, GP(S,Y,G), (GA)n/An, and GGX [7]. Spider silk proteins, composed of glycine-rich sequences with amorphous domains [6], exhibit the tensile properties of polymer fibers. The egg case filaments, abundant in serine [8], possess superior wettability and moisturizing properties [9]. Spider silk’s unique properties are offering a major trend in textile advancements [10]. Spider egg case filaments are biocompatible and antimicrobial, with potential applications in drug delivery, tissue engineering, and infection prevention [11,12]. Furthermore, they possess numerous other advantages, and many aspects of their properties and uses remain to be explored.

Spider silk possesses excellent properties and holds a wide range of potential applications [13]. However, the mechanical properties of natural spider silk fibers are easily affected by individuals and the environment, showing instability, and spiders are also predatory, making it difficult to domesticate them and collect large amounts of spider silk. At present, artificial spider silk is mainly derived from genetically modified eukaryotes in silkworms, prokaryotes in Escherichia coli, etc. Physical and chemical methods to obtain artificial spider silk are mainly concentrated in spider silk protein modification, spinning process alterations, ultraviolet radiation, and other spinning solutions, composite spinning, etc. [14]. However, the artificial synthesis of spider silk via physicochemical methods consumes significant amounts of manpower and material resources, whereas genetic modification offers a means to reduce costs and produce sustainable, stable hereditary spider silk.

Silkworms possess not only economic and practical value but also serve as an ideal model organism for studying eukaryotic expression systems [15]. As one of the earliest animal fibers utilized by humans, silk shows excellent mechanical properties, biocompatibility, and biodegradability [16]. Also, it does not cause immune rejection and toxic side effects in organisms [17], making it valuable in diverse fields such as silk fabrics and medical drugs [18]. Transgenic silkworms can reduce costs by steadily producing stable exogenous proteins for long periods. They also allow researchers to study gene expression, regulation, and function in eukaryotes more deeply. Additionally, the introduction of exogenous genes can positively impact the genetic traits of silkworms [19].

The *piggy*Bac transposon, a DNA transposon with both randomness and preference [20,21,22,23], was first applied to silkworms by Tamura et al. [24]. Subsequently, Thomas et al. [25] improved the process to obtain a better expression effect. Additionally, *piggy*Bac transposons are known for their versatility as vectors, capable of carrying long gene fragments. They can be randomly inserted into the silkworm genome, expressed stably, and inherited, thereby fully harnessing the potential of silkworms in the field of bioreactors [26]. Teulé, F et al. [27] have developed a new transgenic silkworm capable of spinning fibers containing spider silk protein. Tomita et al. [28] expressed traction silk proteins of varying molecular weights in the silk glands of transgenic silkworms. Xu et al. [29] introduced *Nephila* spiders’ MaSp1 traction silk gene into silkworms using TALEN technology. Mi et al. [30] used CRISPR/Cas9 to produce full-length spider silk proteins in silkworms. In this study, *piggy*Bac-mediated transgenic technology was used to express the egg case filament protein of *Nephila clavata*, facilitated by the silkworm heavy-chain promoter, aiming to improve the initial modulus and hygroscopicity of silk.

## 2. Results

### 2.1. Optimize the Sequence of the Gene of Interest

**Figure 1:**
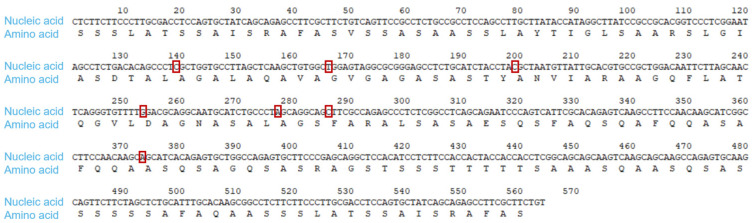
Optimally designed spider egg case silk: basic unit.

According to the cDNA sequences of two complete spider egg case filaments of *Nephila clavata* reported by Zhao [7] in 2005, we obtained the gene sequence numbers for the spider egg case filaments: the 3′ end was AB218973 and the 5′ end was AB218974. Searching the gene sequence information from the NCBI, seven repeat fragments were selected, and a representative gene sequence with a length of 564 bp named *Nc*-CYSP1 was designed and compared; the corresponding amino acid sequence consisted of 187 amino acids. Based on the comparison of the gene sequences of the repeat fragments of the spider egg case filaments, the bases were optimized. Base 139 was originally T, optimized to C; Base 166 was originally C, optimized to T; Base 199 was originally T, optimized to C; Base 253 was originally A, optimized to G; Base 277 was originally G, optimized to A; Base 286 was originally T, optimized to C; Base 373 was originally T, optimized to A (Figure 1).

### 2.2. piggyBac Vector Design and Construction

**Figure 2:**
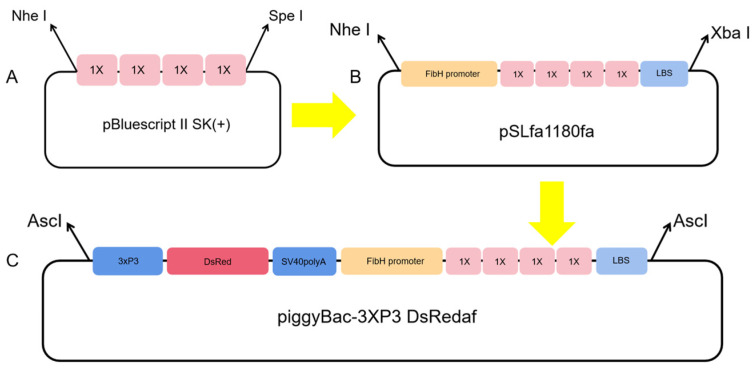
Schematic diagram of vector construction: (**A**) the cloning vector with quadruple target genes; (**B**) the shuttle vector with a quadruple gene of interest; and (**C**) the *piggy*Bac transposable vector with the quadruple target gene.

**Figure 3:**
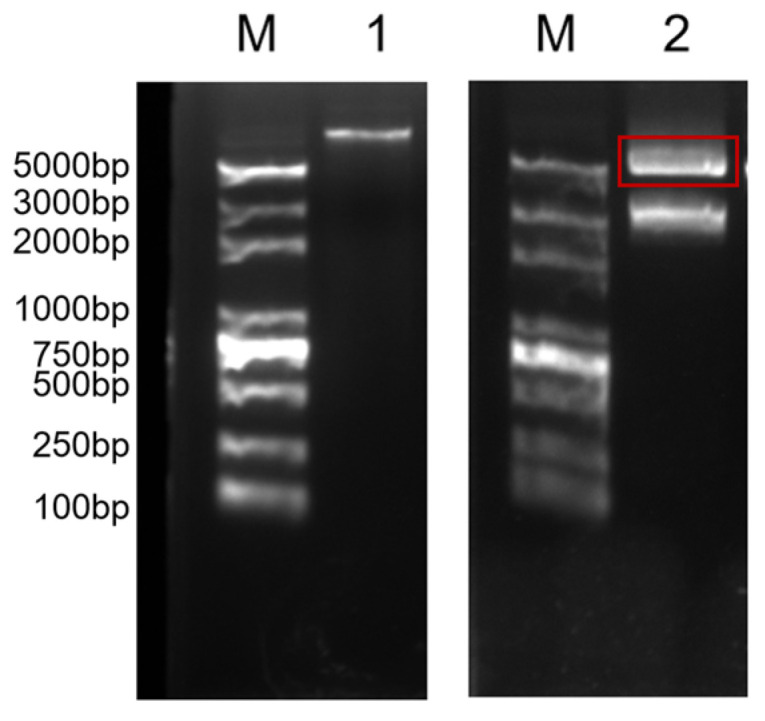
Vector pBAC-3xP3-DsRed-FibH-*Nc*-4xCYSP1-LBS and enzyme digestion validation. M: DL5000Marker; 1: vector pBAC-3xP3-DsRed-FibH-*Nc*-4xCYSP1-LBS; 2: vector pBAC-3xP3-DsRed-FibH-*Nc*-4xCYSP1-LBS enzyme digestion.

Fragments containing 4-fold, 8-fold, and 16-fold sequences of the target gene *Nc*-CYSP1 were ligated to obtain the corresponding p-Bluescript II SK (+)-*Nc*-CYSP1 cloning vector, and electrophoresis verified that the plasmid sizes matched the expected plasmid size. The target gene fragments of different folds were then doubly digested and linked to the engineered shuttle vector pSLfa1180fa-FibH, and the expression was terminated by the LBS sequence that was linked to the tail. The double digestion of target gene fragments (FibH-*Nc*-CYSP1-LBS) of different folds was ligated with the *piggy*Bac vector (Figure 2). Due to the high-fold target gene fragment being too large, only the 4-fold target gene fragment was successfully ligated with the *piggy*Bac vector, and the expression vector pBAC-3xP3-DsRed-FibH-*Nc*-4xCYSP1-LBS was obtained (Figure 3).

### 2.3. Silkworm Transformation

A solution containing 10 μL of the plasmid pBAC-3xP3-DsRed-FibH-*Nc*-4xCYSP1-LBS mixed with an equal volume of the coenzyme plasmid hsp was used for injection, and the results are summarized in Table 1. Approximately 640 silkworm eggs (G0) were injected, and two silkworms in the G1 generation had obvious red fluorescence; one of the fifth-instar silkworms failed to cluster successfully, and the other silkworm successfully emerged as a male moth. The fluorescent male silkworm was mated with a non-injected D9L female, producing four batches of eggs. Most glowed under a fluorescence microscope. We reared the positive ones (G2) for future tests.

**Figure 4:**
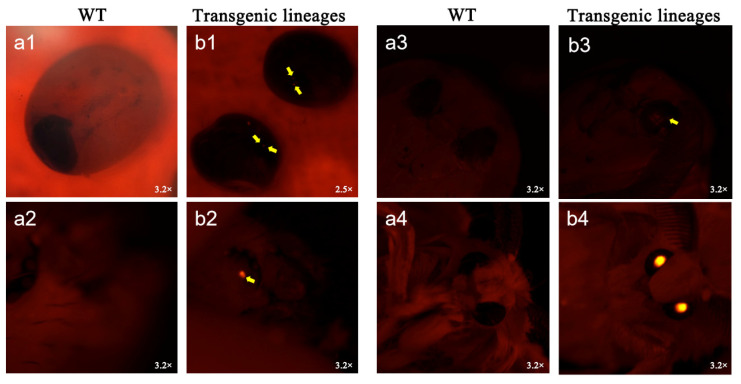
Comparison chart of a transgenic positive silkworm moth in various periods; wild-type WT and G1 egg (**a1**,**b1**), larval (**a2**,**b2**), pupal (**a3**,**b3**), and moth (**a4**,**b4**) stages, respectively, were observed under red fluorescence. It can be observed that only transgenic positive silkworms can specifically express the DsRed gene at the egg, larval, pupal, and moth stages and red fluorescence in the eyes.

Under fluorescence microscopy, transgenic silkworms and wild-type silkworms showed significant morphological differences at different growth stages (Figure 4). The transgenic silkworms displayed distinct red fluorescence under fluorescence microscopy at the egg, larval, pupal, and moth stages, whereas the wild-type non-transgenic silkworms did not exhibit such fluorescence characteristics. This observation indicates that the DsRed red fluorescent protein gene was successfully introduced and expressed in silkworms. However, further detection is still required to confirm whether the target gene *Nc*-4xCYSP1 has been successfully inserted into the silkworm genome and expressed.

### 2.4. Inverse PCR Analysis

**Figure 5:**
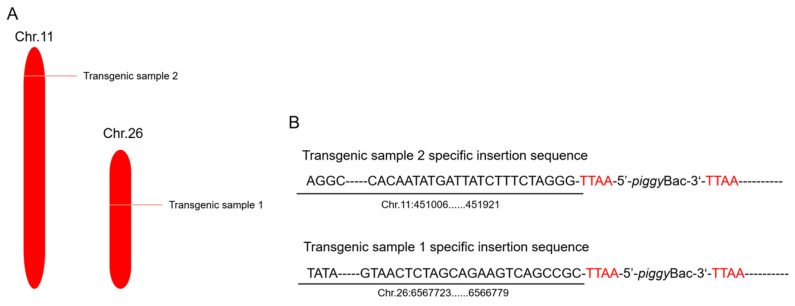
Insertion site analysis of transgenic silkworms. (**A**) Distribution of insertion sites of transgenic silkworms. (**B**) Specific insertion sequences of transgenic silkworms.

The *piggy*Bac transposon system can be randomly inserted into any position in the genome, although it also exhibits a certain preference for inserting into specific sites. It allows for the insertion of any chromosome at an arbitrary position in the genome where the *piggy*Bac system recognizes the TTAA sequence. In this study, the transgenic silkworm genes were extracted, inverse PCR was performed, and the PCR products were ligated to the T vector and sequenced. Through sequencing, the sequences of the right arm of the *piggy*Bac transposon system were obtained. These sequences were subsequently compared with the KAIKObase database. The comparison results revealed that the two transgenic silkworms were inserted into the silkworm genome at different locations, on chromosome 11 and chromosome 26, respectively (Figure 5). Although the inserted foreign genes were identical, the location of the insertions in the genome was different, providing strong evidence for the randomness of *piggy*Bac transposon movement within genomic DNA.

### 2.5. Quantitative Real-Time PCR (qRT-PCR) Analysis

**Figure 6:**
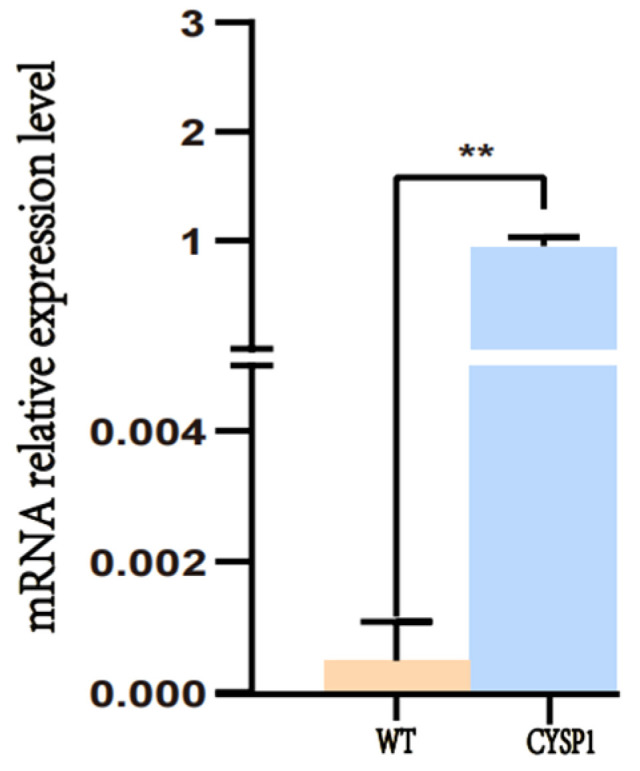
Results of relative mRNA expression. WT: D9L genetically unmodified silkworm. CYSP1: Genetically modified silkworms. Three biological replicates per sample, using the two-tailed test mode: ** means *p* < 0.01 and * indicates *p* < 0.05.

To detect whether the egg case filament protein of *Nephila clavata* was overexpressed in silkworms, a pair of specific primers was designed for the target gene sequence using Primer 5 software, and the transcription level of the exogenous protein was detected via real-time PCR (qRT-PCR). RNA from posterior silk glands of G2 wild-type and transgenic silkworms (on the third day of five instars) was extracted and reverse-transcribed to cDNA. Using this cDNA as the template, fluorescence quantitative PCR was performed with BmRpl3 as an internal control and wild-type D9L non-transgenic silkworms as negative controls. The results showed that a very small amount of expression was detected in the control variety, while a large number of related genes could be detected in the transgenic line, differing significantly from the wild type (*p* < 0.01). The results, shown in Figure 6, indicate that the exogenous gene of the *Nephila clavata* spider egg case silk protein was successfully transcribed in silkworms, and the transcription level was high.

### 2.6. SDS-PAGE

**Figure 7:**
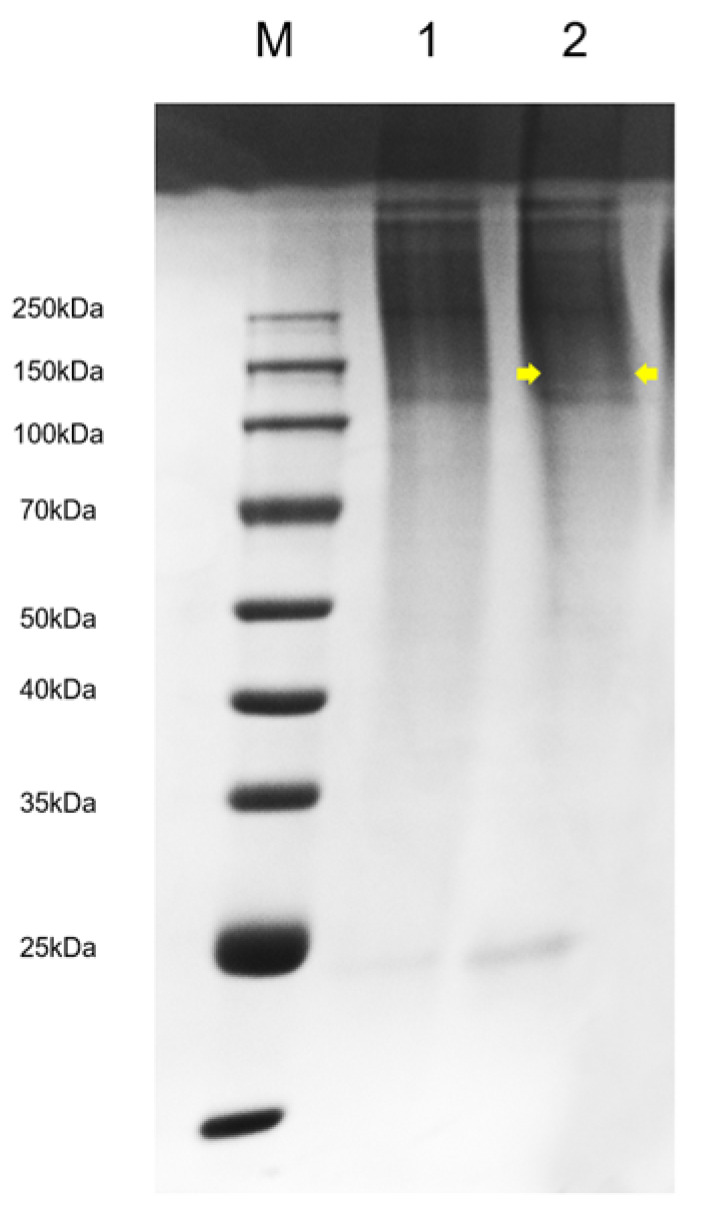
SDS-PAGE protein electrophoresis. M: protein molecular weight. 1: sample of common D9L silkworm cocoon protein. 2: A sample of silkworm cocoon protein from the egg case silk gene of *Nephila clavata*.

In order to detect whether the egg case filament protein of the *Nephila clavata* can be normally expressed in the posterior silk gland of transgenic silkworms and secreted into the cocoon, the transgenic silkworm cocoons were taken and dissolved in 10 mol/L lithium thiocyanate solution to extract the protein. SDS-PAGE protein electrophoresis was performed; subsequently, Coomassie brilliant blue staining was performed and then decolorized. Through comparison with the common D9L silkworm cocoon, it was found that there were obvious specific bands in the 100 kDa band, indicating that the egg case filament protein of *Nephila clavata* was successfully expressed in the silkworm’s posterior silk gland and secreted into the cocoon (Figure 7). Using the BCA kit, we measured the concentration of the lysed protein samples by determining their absorbance (A562) at 562 nm with a microplate reader. The calculations showed that the solubility concentration of the common D9L silkworm cocoon protein was 49.72 mg/mL, whereas the concentration of the silkworm cocoon protein derived from the *Nephila clavata* egg case filament protein was 44.20 mg/mL.

### 2.7. Amino Acid Analysis

The amino acid content of silk protein is crucial in determining its secondary structure, which ultimately influences the quality and mechanical properties of silk fibers. Table 2 presents the proportions of amino acids in the silk fibroins and whole cocoons of ordinary silkworm cocoons and genetically modified silkworm cocoons, as well as the *Nephila clavata* egg case silk. Upon comparison, it was observed that there were increases in the content of both acidic and basic amino acids. This alteration has the potential to modify the protein’s secondary structure, thereby inducing changes in the interactions within its tertiary structure. Additionally, when comparing the proportion of non-polar amino acids, it was found that the total quantity of polar amino acids had increased. This increase facilitates enhanced binding with polar molecules, including water molecules, ultimately leading to an improvement in the water absorption capabilities of silk.

The analysis reveals that the amino acid composition of transgenic silk differs slightly from that of ordinary silk, and there are significant differences compared to the egg case silk of *Nephila clavata*. The introduction of exogenous genes has evidently altered the amino acid content of silk. The contents of amino acids that influence the structure of silk, such as alanine, glycine, and tyrosine, have decreased, whereas the contents of serine, glutamic acid, arginine, and proline have increased. Notably, the transgenic silk exhibits increased serine (by 0.28% in silk fibroin and 0.15% at the cocoon level) and proline (by 0.06% in silk fibroin and 0.41% at the cocoon level) compared to ordinary silk. This suggests the successful incorporation of spider silk genes into the silkworm genome, altering the amino acid profile of the silk. The increased serine and proline content in the transgenic silk is likely to have implications for its water absorption capacity and the proportion of β-turns in its secondary structure, which are key factors influencing the physicochemical properties of silk. Consequently, the modified amino acid composition of the transgenic silk is expected to lead to significant changes in its physical and chemical properties compared to ordinary silk.

### 2.8. Scanning Electron Microscopy

**Figure 8:**
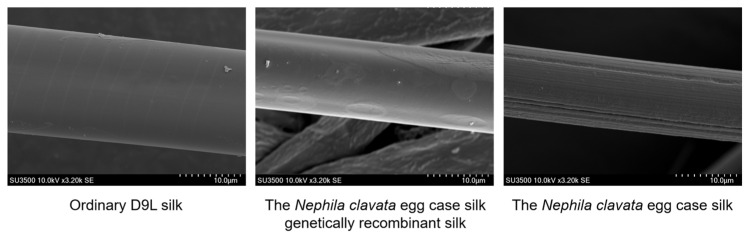
Scanning electron microscope (SEM) images of silk fibers.

Scanning electron microscopy (SEM) showed that the surface of the silk gene between ordinary D9L silk and the transgenic silk was relatively smooth, and there were no significant differences. Unlike the silk structure, the morphology of the spider silk fibers observed under electron microscopy was different, and the surface of the egg case filament protein of *Nephila clavata* was observed to be rough (Figure 8).

The diameters of the three samples were measured and calculated, and it was found that the diameter of ordinary D9L silk was greater, while there was no significant difference in diameter between the genetically recombinant *Nephila clavata* egg case silk and the normal *Nephila clavata* egg case silk. During the fifth instar, individual transgenic silkworms were observed to be smaller compared to ordinary silkworms, and their silking speed was also slower. Analysis showed that this may have been due to the high molecular weight of the spider silk protein itself, which caused the silkworms difficulties in silking. The electron microscope image reveals significant variations in *Nephila clavata* egg case silk thickness, and it confirms the presence of both thick and thin filaments. The diameters were 18.2 ± 0.4 μm for ordinary D9L silk, 13.0 ± 0.8 μm for genetically recombinant *Nephila clavata* egg case silk, and 12.6 ± 0.6 μm for thick *Nephila clavata* egg case silk.

### 2.9. Fourier-Transform Infrared Spectroscopy

**Figure 9:**
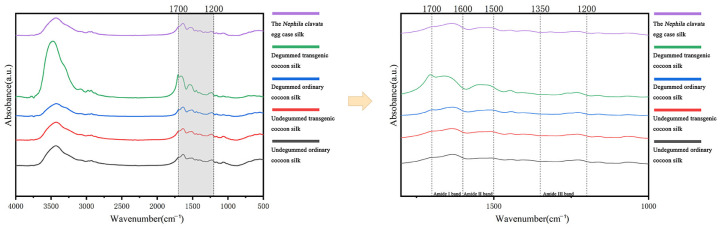
FTIR results of silks.

In order to further analyze the effects of exogenous genes on the mechanical properties of silk, Fourier-transform infrared spectroscopy was carried out on the undegummed ordinary cocoon silk, undegummed transgenic cocoon silk, degummed ordinary cocoon silk, degummed transgenic cocoon silk, and *Nephila clavata* egg case silk, and the secondary protein structure of the above fiber samples was characterized and analyzed. From the absorption rate in the wavelength range of 4000–500 cm^−1^, it can be seen that there are three main characteristic peaks of amide groups, i.e., the amide I band (1700–1600 cm^−1^), amide II band (1600–1500 cm^−1^), and amide III band (1300–1200 cm^−1^) and the characteristic peaks of the infrared absorption spectra of the five samples are not significantly shifted (Figure 9).

**Figure 10:**
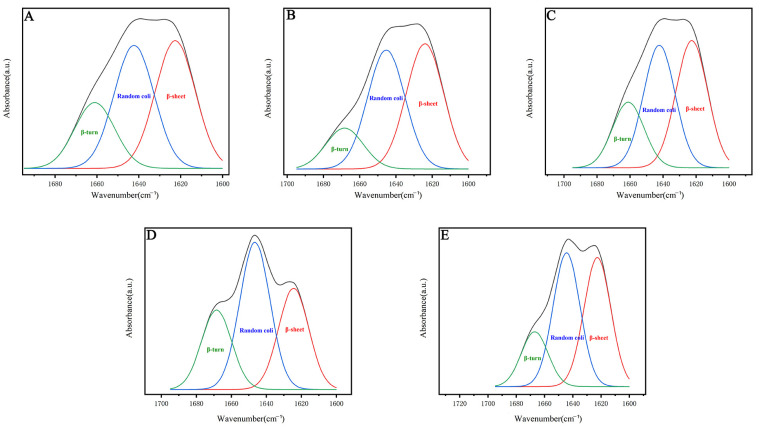
Infrared spectroscopy of amide I band peak-fitting results. (**A**) Undegummed cocoon silk. (**B**) Undegummed transgenic cocoon silk. (**C**) Degummed common cocoon silk. (**D**) Degummed transgenic cocoon silk. (**E**) The egg case silk filament protein from *Nephila clavata*.

The amide I band (1700–1600 cm^−1^) was mainly fitted by peak, and the main characteristic peaks of the secondary protein structure were analyzed and found as follows: β-sheets at 1600–1640 cm^−1^, random curls and α-helices at 1640–1660 cm^−1^, and β-turns at 1660–1700 cm^−1^. No significant change was observed in the position of the main characteristic peaks, which suggests that the introduction of exogenous genes did not alter the basic structure of the silk. Upon further analysis of the amide I band split (Figure 10), Table 3 shows transgenic silk, both undegummed and degummed, has higher β-turns and random curls/α-helices than ordinary silk. The degumming process further enhances these structural changes in transgenic silk. Concurrently, *Nephila clavata* egg case silk exhibits a superior secondary structure with more β-turns and random curls/α-helices than undegummed common cocoon silk. This also suggests that spider silk, which inherently has a lower β-sheet content compared to silk, compensates for the absence of β-sheets in its protein structure by possessing a flexible secondary structure that significantly contributes to the elongation of silk fibers, and spider silk protein has a larger amorphous domain [31,32], which makes it malleable and elastic. Also, the presence of serine residues forms more hydrogen bonds, improving the structural stability of spider silk proteins. In summary, the integration of exogenous proteins into *Nephila clavata* egg case silk significantly modified its secondary structural composition, ultimately altering the mechanical properties of the silk fibers, validating the experimental hypotheses.

### 2.10. Mechanical Testing of Transgenic Silk Fibers

**Figure 11:**
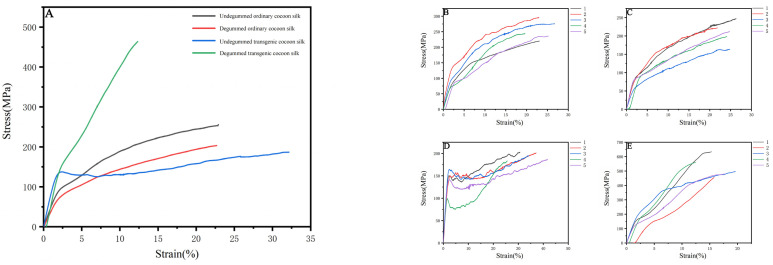
Stress–strain curves of different silk strains: (**A**) all silk strains, (**B**) undegummed cocoon silk, (**C**) degummed cocoon silk, (**D**) undegummed transgenic cocoon silk, and (**E**) degummed transgenic cocoon silk.

**Figure 12:**
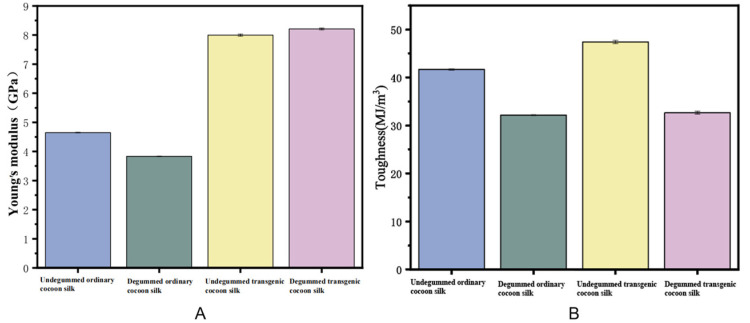
Histograms of the (**A**) Young’s modulus and (**B**) toughness of different silk strains.

A universal tensile testing machine was used to detect the differences between degummed/undegummed ordinary silk and genetically modified silk under the same conditions. Meanwhile, we consulted literature and introduced *Bombyx mori* silk and spider egg case silk (specifically *Araneomorpha’*s egg case silk, as we did not find available mechanical performance data for *Nephila clavata*) as references. Through comparison, we also identified significant differences in the mechanical properties between *Bombyx mori* silk and D9L silk. According to the analysis of the mechanical properties statistics (Table 4) and the stress–strain curve (Figure 11), degummed transgenic silk had a breaking strength 260.8 MPa higher than degummed ordinary silk. Compared with ordinary silk, both undegummed and degummed transgenic silk exhibited increased Young’s modulus, by 3.348 GPa and 4.378 GPa, respectively, as well as improved toughness, by 5.738 MJ/m^3^ and 0.497 MJ/m^3^, respectively. However, the breaking strain of degummed transgenic silk decreased by 10.351% compared to degummed ordinary silk. In comparison to ordinary silk, the transgenic silk exhibited distinct yield points, suggesting that the hydrogen bonds within it underwent significant breaking and reforming during stretching, attributed to the presence of different molecules. In summary, the addition of exogenous proteins significantly enhanced both the Young’s modulus and toughness of transgenic silk, especially the Young’s modulus (Figure 12); this is consistent with the original expectation that the *Nephila clavata* egg case silk protein would improve the elasticity and toughness of silk. However, the results of the undegummed silk’s performance and the breaking strain of degummed transgenic silk were not ideal. Poor winter conditions for silkworms may have reduced the quality of silk spinning. Additionally, these results are also linked to gene duplication in spider silk, large domestic silk proteins, and difficulties in silk synthesis and secretion.

### 2.11. Water Absorption Performance Test

We weighed each of the five samples in each group. For *Nephila clavata* silk, due to its loose form, only 0.01 g was weighed as a reference. The mass before water absorption for all samples was 0.01 g. Due to the individual differences in the quality of the samples, we calculated the average mass of the samples after water absorption to determine the water absorption rate, as analyzed in Table 5.

We observed that genetically modified *Nephila clavata* silk absorbed more water than ordinary silk, and natural *Nephila clavata* silk absorbed even more. The increased serine content in the transgenic silk enhanced its water absorption performance, which was consistent with our expectations. The results confirm that the genetically recombinant *Nephila clavata* egg case silk is capable of improving silk’s water absorption properties.

## 3. Discussion

In this study, we used silkworms as bioreactors with a eukaryotic expression system to analyze *Nephila clavata* egg case protein genes, achieving the expression of its functional protein and novel transgenic spider silk materials. The transgenic silkworm expression vector based on the *piggy*Bac transposase system was successfully constructed, and two positive silkworm lines were obtained via microinjection; the positivity rate was 0.05%. Due to the randomness and preference of the *piggy*Bac transposon, the chromosome positions inserted into the two transgenic silkworms were also different (chromosomes 11 and 26, respectively). Compared with ordinary silkworms, the mRNA expression level in the posterior silk glands of transgenic silkworms was higher. The expression of the cocoon layer protein was confirmed by the appearance of specific bands, which indicated the successful transfer and expression of the exogenous *Nephila clavata* egg case silk protein gene in the silkworms. In comparison with ordinary silk, transgenic silk exhibited no notable differences in terms of appearance and morphology, with the exception of a slightly smaller diameter. The analysis of amino acid contents revealed an increase in serine and proline levels, resulting in enhanced water absorption capabilities of the silk and an augmentation in the number of β-turns within its secondary structure. From the perspective of mechanical properties, the proportion of random coils/α-helices and β-turns in the secondary structure of the transgenic silk protein increased, increasing the elongation performance of the silk. The initial modulus of the transgenic silk was increased by 4.738 GPa, and its toughness was increased by 5.738 MJ/m^3^. Due to the increase in serine content, the water absorption performance of the transgenic silk was also increased by 0.74% compared with that of ordinary silk.

## 4. Materials and Methods

### 4.1. Animals

In this experiment, the D9L silkworm variety was used, which was preserved in the laboratory and pluralized, with yellow cocoons; each generation spanned ~45 days. The silkworms were reared with fresh mulberry leaves at 25 °C and 75% relative humidity.

### 4.2. piggyBac Vector Construction

In this study, seven repeats in the egg case filament protein CySp1 gene of *Nephila clavata* were compared, integrated, and optimized, and an *Nc*-CYSP1 gene unit with a length of 564 bp was designed and synthesized. The gene units were multiplied several times to construct gene fragments containing 4-fold, 8-fold, and 16-fold repetitions of target gene unit sequences. Different folds of the target gene were ligated to the modified shuttle vector pSLfa1180fa (constructed in our laboratory), and the silkworm heavy-chain promoter was added in front of the target gene; LBS was added to the 3′ end to terminate expression. Finally, the modified 4-fold target gene was selected for follow-up experiments. The gene fragment, after being digested with the restriction enzyme ASC I, was linked to the vector pBac-[3xp3-DsRed] (constructed in our laboratory) and named pBAC-3xP3-DsRed-FibH-*Nc*-4xCYSP1-LBS.

### 4.3. Silkworm Transformation

The D9L silkworm eggs laid within 3-4 h were selected for microinjection. The plasmid pBAC-3xP3-DsRed-FibH-*Nc*-4xCYSP1-LBS and the auxiliary enzyme plasmid hsp were mixed and injected into the 1/3–1/4 position of the silkworm eggs at a 1:1 ratio (both concentrations were 700 ng/μL) using a microinjection instrument (Eppendorf, Hamburg, Germany). The injected silkworm eggs were then quickly sealed with non-toxic sealing glue to avoid contamination, sterilized with diluted formaldehyde for 5 min, and placed in the incubator at 25 °C for moisturizing incubation.

### 4.4. Inverse PCR Analysis

In order to determine the insertion site information of the transgenic exogenous protein CYSP1 gene in silkworms, the previously extracted genomic DNA of silkworms was used as a template for inverse PCR. The genomic DNA of silkworms was linearized by enzymatic digestion using the restriction enzyme Hae III, then cyclized using T4 ligase (TaKaRa, Beijing, China), the ligation product was used as a template, PCR amplification was carried out, T-A cloning was performed after product recovery, and PMD19-TSimple was ligated. The sequencing results were performed on the silkworm genome KAIKO (https://kaikobase.dna.affrc.go.jp/index.html (accessed on 24 November 2024)) to determine the precise location of the insertion site.

### 4.5. Quantitative Real-Time PCR Analysis

To detect whether the spider egg case filament protein of *Nephila clavata* was overexpressed in silkworms, a pair of specific primers were designed for the target gene sequence using Primer 5 software, and the transcription level of the exogenous protein was detected via fluorescence quantitative PCR (qRT-PCR). RNA was extracted from the posterior silk glands of fifth-instar, three-day-old wild-type (G2) silkworms and their transgenic counterparts. This RNA was then reverse-transcribed into cDNA. Using this cDNA as the template, BmRpl3 as an internal control, and wild-type D9L non-transgenic silkworms as controls, fluorescence quantitative PCR experiments were carried out. Three biological replicates per sample were analyzed using a two-tailed test mode.

### 4.6. SDS-PAGE

A total of 0.25 g of silkworm cocoon/spider egg case filament was processed by cutting it finely and dissolving it in 0.5 mL of 10 mol/L lithium thiocyanate at 60 °C for 12 h. It was then centrifuged at RT, 15,000 rpm for 5 min. The supernatant was collected, the pellet was discarded, and the supernatant was stored at −20 °C. Protein samples were mixed with 2× loading buffer, denatured at 100 °C for 10 min. Gels were prepared using the PAGE gel electrophoresis kit (Epizyme, Shanghai, China). After adding the sample, electrophoresis was conducted at 150 V, 200 mA for approximately 1.5 h. This was followed by staining with Coomassie blue, decolorizing, and photographing for analysis.

### 4.7. Amino Acid Analysis

A certain amount of ordinary cocoon silk and transgenic cocoon silk were taken and placed into a 0.5% Na_2_CO_3_ solution at 100 °C, degummed at a liquor ratio of 1:50 for 30 min, and the degummed silk fiber was washed with ddH_2_O 3 times and dried for subsequent testing. Separately, 80 mg of each of undegummed silk, degummed silk, and spider egg case filament fiber materials were taken and shredded, and the hydrolyzed amino acids were measured by an automatic amino acid analyzer (Biochrom, Cambridge, UK).

### 4.8. Scanning Electron Microscope

We took appropriately sized samples of D9L silk, transgenic silk, and the *Nephila clavata* egg case silk and fixed them to the stage using conductive tape. The stage was placed in a vacuum sputterer, and the surface of the samples was gold-plated at 25 °C and 15 kV. Under standard flow beam conditions of 10 kV, photographs were acquired using a field-type scanning electron microscope (Phenom, Rotterdam, The Netherlands). Three samples of each silk were randomly selected for observation, and from each sample, five silks were separately chosen for measurement.

### 4.9. Fourier-Transform Infrared Spectroscopy

After shredding D9L silk, transgenic silk, and *Nephila clavata* egg case silk, 0.005 g of each sample was taken and mixed with 0.5 g of spectral potassium bromide (ratio ~1:100). The resulting mixtures were thoroughly ground, oven-dried, and subsequently pressed into tablets for analysis. A Fourier-transform infrared spectrometer (Thermo, St. Bend, OR, USA) was used to scan the sample spectrum 32 times with a scanning range of 4000–500 cm^−1^, and a resolution of 4 cm^−1^. We collated data and drew the infrared spectrum (ranging from 4000 to 500 cm^−1^) in Origin. We analyzed the amide I band (1600–1720 cm^−1^) using PeakFit(v4.12), fitting peaks and comparing secondary protein structure contents: random coils, α-helices, β-sheets, and β-turns.

### 4.10. Mechanical Testing of Transgenic Silk Fibers

We used A4 paper to make a paper frame for pasting the silk samples, cutting a 4 cm × 2 cm rectangle from the center of an 8 cm × 4 cm piece of A4 paper. We pretreated the silk by removing the outer cocoon layer, soaking the cocoons in warm water, and drawing the silk to obtain undegummed silk samples. To obtain degummed silk, we boiled the cocoons for 5 min, stripped them, and dried the silk. Using double-sided tape, we fixed the ends of the silk to the paper frame. We then tested the mechanical properties of ordinary D9L silk and genetically modified silk using a high-precision desktop tensile testing machine (Shimadzu, Japan). For each type of silk, 30 silk samples were evaluated. Prior to testing, we trimmed off the excess paper frame, set the tensile speed at 10 mm/min, and calculated the average stress and strain values after applying tension.

### 4.11. Water Absorption Performance Test

In order to explore the water absorption of silk with the *Nephila clavata* egg case filament protein gene, we adopted the test method for water absorption rate outlined in GB/T 8939-2018. Sanitary absorbent pads (panty liners) were used as a reference [35]. We separately cut 5 samples (including undegummed ordinary D9L silk, degummed ordinary D9L silk, undegummed transgenic silk, degummed transgenic silk and the *Nephila clavata* egg case silk) with similar size and weight to 0.01 g. At a temperature of 23 ± 1 °C, we took each sample with tweezers and immersed it in ddH_2_O at a depth of 10 cm for 60 s, pressing gently. After this period, we removed the sample, hung it vertically for 90 s to allow excess water to drip off, and then measured its mass to three decimal places.

(1) $$\mathrm{Water}\;\mathrm{absorption}\;\mathrm{rate}=\frac{\mathrm{Mass}\;\mathrm{after}\;\mathrm{water}\;\mathrm{absorption}-\mathrm{Mass}\;\mathrm{before}\;\mathrm{water}\;\mathrm{absorption}}{\mathrm{Mass}\;\mathrm{before}\;\mathrm{water}\;\mathrm{absorption}}$$

## 5. Conclusions

This study represents the first time that the egg case filament protein gene of *Nephila clavata* has been transferred into silkworms and expressed. The results show that the exogenous protein gene was successfully transferred and effectively improved the performance of silk fibers. This provides a lot of material for more in-depth research on the egg case filament protein of *Nephila clavata*, promoting the exploration of functional silk materials with silk modification and new transgenic spider silk genes.

## Figures and Tables

**Table 1:** Genetically modified silkworm injection results.

Genetically Modified Silkworm Strains	Transgenic Plasmid Concentration (ng/μL)	Coenzyme Plasmid hsp Concentration (ng/μL)	Number of Eggs Injected (Grains)	Number of Eggs Hatched (Grains)	Hatchability (%)	Number of Moth Areas in the G1 Generation	Number of Positive Moth Areas in the G1 Generation	Positivity Rate (%)
*Nc*-4xCYSP1	716.5	719.5	640	26	4%	20	1	5%

**Table 2:** Amino acid composition analysis.

Relative Amino Acid Contents (%)	Ordinary Silkworm Cocoon		Genetically Modified Silkworm Cocoon		Egg Case Silk of *Nephila clavata*
	Degummed Silk	Undegummed Silk	Degummed Silk	Undegummed Silk	Egg Case Silk
Aspartic acid	2.59	7.46	2.73	7.92	4.79
Threonine	1.38	3.47	1.41	3.54	2.78
Serine	13.13	16.34	13.41	16.49	9.87
Glutamic acid	2.36	3.41	2.28	3.47	15.74
Glycine	26.36	21.35	25.94	21.22	13.51
Alanine	28.07	21.33	27.91	19.88	16.67
Cystine	3.57	3.37	3.49	3.62	3.65
Valine	3.53	3.50	3.60	3.47	3.48
Methionine	0.62	0.51	0.57	0.48	0.85
Isoleucine	0.70	0.77	0.78	0.76	1.65
Leucine	0.76	1.00	0.82	1.06	5.93
Tyrosine	12.70	10.28	12.59	9.83	6.22
Phenylalanine	1.53	1.50	1.62	1.78	2.69
Histidine	0.55	1.06	0.54	1.28	1.09
Lysine	0.56	1.67	0.58	1.76	2.68
Arginine	0.81	2.15	0.89	2.28	4.41
Proline	0.78	0.83	0.84	1.24	4.05
**Acidic amino acids**	4.95	10.87	5.01	11.39	20.53
**Basic amino acids**	1.92	4.88	2.01	5.32	8.18
**Polar amino acids**	37.65	49.21	37.92	50.19	51.23
**Non-polar amino acids**	62.35	50.79	62.08	49.89	48.83

**Table 3:** Analysis of the secondary structure content of the silk fibers.

Sample	β-Sheets (1600–1640 cm^−1^)	Random Curls/α-Helices (1640–1660 cm^−1^)	β-Turns (1660–1700 cm^−1^)
Undegummed ordinary cocoon silk	44.25%	41.57%	14.18%
Undegummed transgenic cocoon silk	43.93%	41.73%	14.33%
Degummed ordinary cocoon silk	40.33%	38.87%	20.80%
Degummed transgenic cocoon silk	30.87%	44.91%	24.21%
*Nephila clavata* egg case silk	40.63%	42.12%	17.26%

**Table 4:** Mechanical properties of cocoon silk, mulberry silk, and natural egg case silk.

Sample	Strength (MPa)	Elongation (%)	Young’s Modulus (GPa)	Toughness (MJ/m^3^)
Undegummed ordinary cocoon silk	255.664 ± 40.336	22.908 ± 3.812	4.650 ± 0.1455	41.673 ± 14.697
Degummed ordinary cocoon silk	203.112 ± 44.074	22.691 ± 3.633	3.833 ± 0.0924	32.176 ± 13.514
Undegummed transgenic cocoon silk	186.936 ± 16.178	32.169 ± 9.500	7.998 ± 0.3392	47.411 ± 18.561
Degummed transgenic cocoon silk	463.912 ± 8.515	12.340 ± 7.271	8.211 ± 0.2878	32.673 ± 38.707
*Bombyx mori* silk (mulberry silk) [33]	500–600	18	9.600 ± 0.6000	70
Egg case silk (*Araneomorpha*) [34]	400 ± 50	5–20	8.700 ± 0.9000	-

**Table 5:** Mass before and after water absorption.

Sample	Mass Before Water Absorption (g)	Mass After Water Absorption (g)		Water Absorption Rate (%)
Ordinary D9L silk	0.010	Sample	Mass (g)	8.46
		1	0.116	
		2	0.057	
		3	0.069	
		4	0.089	
		5	0.092	
		Average	0.0846	
Genetically recombinant *Nephila clavata* egg case silk	0.010	Sample	Mass (g)	9.2
		1	0.116	
		2	0.057	
		3	0.069	
		4	0.089	
		5	0.092	
		Average	0.0920	
*Nephila clavata* egg case silk	0.010	0.0950		9.5

## Data Availability

Data are contained within the article.
